# Echocardiographic monitoring in cancer therapy: clinical guidance for cardiologists and oncologists

**DOI:** 10.1007/s10741-025-10569-0

**Published:** 2025-10-24

**Authors:** Simona Sperlongano, Giuseppe Verde, Natale Guarnaccia, Felice Gragnano, Giovanni Benfari, Vincenzo De Sio, Federica Ilardi, Matteo Lisi, Alessandro Malagoli, Giulia Elena Mandoli, Maria Concetta Pastore, Ciro Santoro, Matteo Cameli, Giovanni Cimmino, Paolo Calabrò, Antonello D’Andrea

**Affiliations:** 1 Division of Cardiology, Department of Translational Medical Sciences, University of Campania Luigi Vanvitelli, Naples, Italy; 2 Section of Cardiology, Department of Medicine, University of Verona, Verona, Italy; 3 Department of Advanced Biomedical Sciences, University of Naples Federico II, Naples, Italy; 4 Department of Cardiovascular Disease - AUSL Romagna, Division of Cardiology, Ospedale S. Maria Delle Croci, Ravenna, Italy; 5 Division of Cardiology, Nephro-Cardiovascular Department, Baggiovara Hospital, University of Modena and Reggio Emilia, Modena, Italy; 6 Department of Medical Biotechnologies, Division of Cardiology, University of Siena, Siena, Italy; 7 Department of Pharmacy, and Health and Nutrition Sciences, University of Calabria, Cosenza, Italy; 8 Department of Cardiology, Umberto I Hospital, Nocera Inferiore, Italy

**Keywords:** Transthoracic echocardiography, Cardio-oncology, Cancer therapy-related cardiac dysfunction, Left ventricular function, Right ventricular function

## Abstract

Transthoracic echocardiography has a central role in the diagnosis and monitoring of cancer therapy-related cardiac dysfunction, offering a reliable, non-invasive, and cost-effective tool for the early detection of myocardial damage and the timely and effective management of cardiotoxicity. This review provides a practical and comprehensive framework for the echocardiographic assessment of oncology patients, focusing on both traditional and emerging ultrasound parameters of left and right ventricular function, and their diagnostic and prognostic value in the field of cardio-oncology. Moreover, the review discusses key aspects of pericardial disease, valvular heart disease, and intracardiac masses, which may be consequences of cancer therapy or tumor progression. Finally, the role of multimodal imaging, in particular cardiac magnetic resonance and computed tomography, is examined, especially in selected cases to supplement echocardiographic findings or when echocardiography presents limitations. Based on current guidelines and clinical experience, this review aims to provide both cardiologists and oncologists with a practical tool for interpreting echocardiographic reports in cancer patients. Additionally, a therapeutic algorithm is proposed to guide decisions on cancer treatment management and timely initiation of cardioprotective strategies in selected contexts. Close collaboration between cardiologists and oncologists remains essential to reduce cancer patients’ cardiovascular risk, allowing them to access the best possible treatment and optimize outcomes by balancing anticancer therapy efficacy with cardiovascular safety.

**Supplementary Information:**

The online version contains supplementary material available at 10.1007/s10741-025-10569-0.

## Introduction

Cancer therapy has significantly improved survival in oncology patients [1]; however, the associated cardiotoxicity remains a major concern, which negatively affects both quality of life and prognosis [2]. The risk of cardiac damage is not the same for all cancer patients, depending on their cardiovascular (CV) profile combined with therapy-related factors, such as drug type and dosage. Early detection and management of therapy-induced cardiotoxicity are essential to mitigate CV complications without compromising treatment efficacy [3]. Echocardiographic evaluation plays a key role in this context, providing a non-invasive and reliable method to monitor cardiac function and detect subclinical dysfunction. In this regard, speckle-tracking derived global longitudinal strain (GLS) has emerged as a highly sensitive marker for identifying early myocardial damage since its impairment often precedes a decline in left ventricular ejection fraction (LVEF) [4, 5]. Effective management of cardiac involvement requires a multidisciplinary approach. Collaboration between cardiologists and oncologists is essential to ensure optimal therapeutic outcomes, balancing cancer treatment efficacy with CV safety [6]. The aim of the present review is to provide both cardiologists and oncologists with a practical tool that can be useful in writing and interpreting echocardiographic reports, respectively. To achieve this goal, key aspects of cardiac systolic and diastolic function, pericardial disease, valvular heart disease, intracardiac masses, and their prognostic value in the field of cardio-oncology are discussed. Moreover, a therapeutic algorithm is proposed, focused on both cancer treatment management and cardioprotective strategies in selected contexts. This guidance may help bridge the gap between the two specialties by providing oncologists with essential insights to effectively integrate CV assessments into their therapeutic decision-making process.

### Cardiotoxicity risk stratification: identification of cancer patients requiring baseline echocardiography

Baseline cardiotoxicity risk stratification represents a crucial step before initiating cancer therapy, as it guides both the appropriate use and optimal timing of echocardiographic evaluation. However, assessing cardiotoxicity risk can be challenging due to multiple confounding factors, including the concomitant use of agents with adverse metabolic effects (e.g., corticosteroids). The 2022 European Society of Cardiology (ESC) guidelines on cardio-oncology [6] support the use of the recently developed Heart Failure Association (HFA) and International Cardio-Oncology Society (IC-OS) baseline risk stratification tool. According to this tool, cardiotoxicity risk is based on the patient’s specific cancer therapy and underlying CV profile (Table S1, Supplementary Material) [7]. Transthoracic echocardiography (TTE) at baseline is recommended for patients with a high or very high CV risk profile or for those receiving cardiotoxic cancer therapies [8]. Individuals with significant valvular heart disease, history of myocardial infarction, prior coronary revascularization, or stable angina, as well as those with baseline LVEF < 50% or age ≥ 80 years, and individuals with pre-existing heart failure or cardiomyopathy are considered respectively at high and very high risk for cardiotoxicity prior to starting anthracycline or human epidermal growth factor receptor 2 (HER2)-targeted therapies. Different classes of cancer therapy are possibly associated with distinct cardiotoxic findings detectable on TTE (Table 1) [2, 9–20]. It should be underlined that CV risk stratification does not aim to exclude high and very high-risk patients from cancer treatment, but to ensure that patients from all risk categories receive the best possible treatment. There is often a tendency to underestimate and undertreat CV risk factors in cancer patients [21]. However, the presence of untreated CV risk factors may potentiate oncology drugs’ cardiac toxicity [22, 23]. Therefore, reducing cancer patients’ CV risk is crucial for them to tolerate cardiotoxic treatments more easily.

**Table 1.**
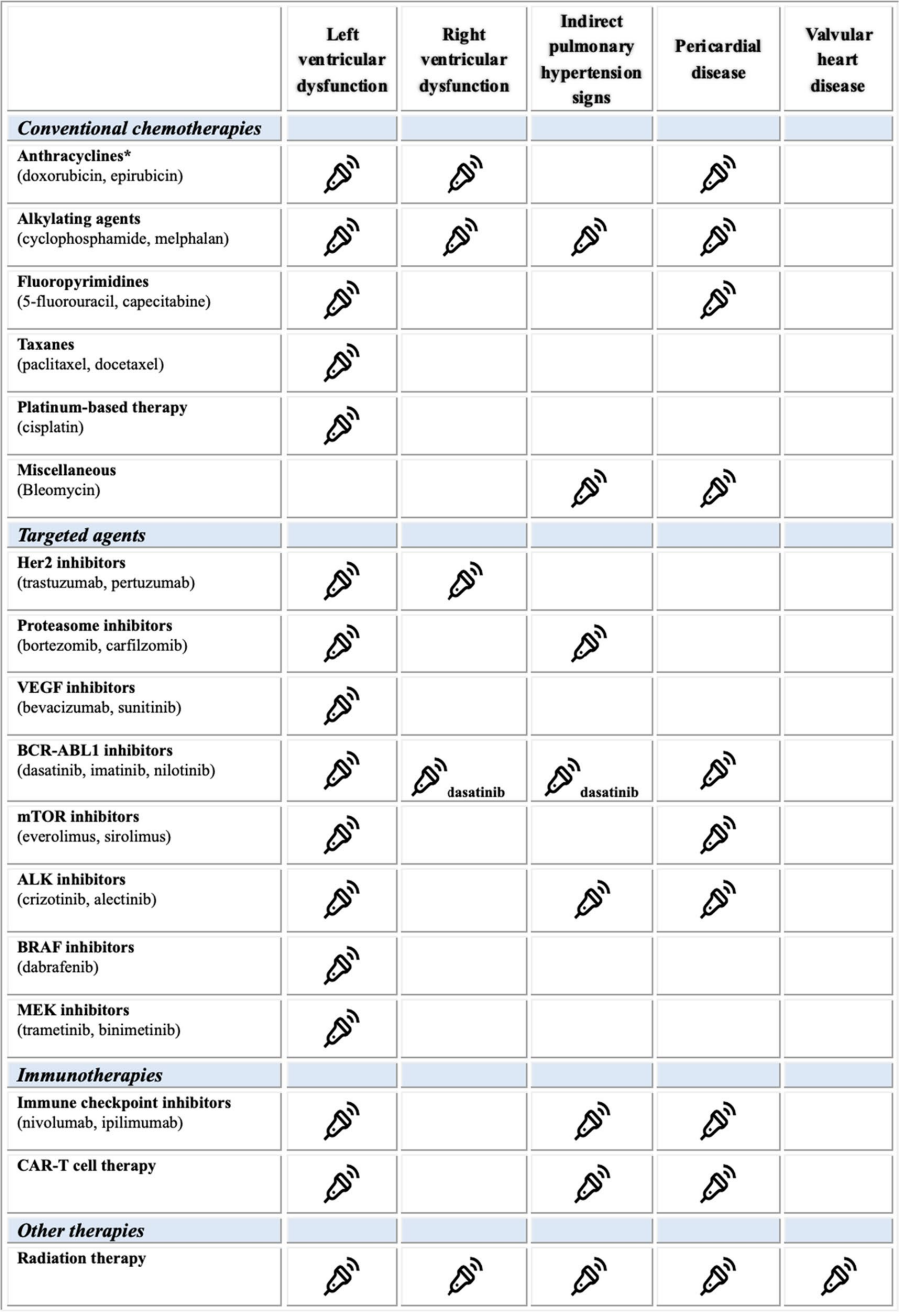
Echocardiographic findings associated with different classes of cancer therapies

*Anthracycline-induced toxicity is a dose-dependent process; therefore, cumulative exposure should be considered in risk assessment

### Chemotherapy-related left heart dysfunction: key echocardiographic parameters and their interpretation

Cancer therapy can adversely affect cardiac structure and function, leading to either asymptomatic cardiac dysfunction or symptomatic heart failure (HF). This condition is collectively termed cancer therapy-related cardiac dysfunction (CTRCD). The most recent definition of CTRCD, derived from the 2022 IC-OS consensus statement [24], is based on changes in LVEF, systolic dysfunction, and/or HF events. LVEF assessed by biplane Simpson’s method remains the most commonly used parameter for the identification of left ventricular (LV) dysfunction before, during, and after cancer therapy. However, it is important to acknowledge the inherent limitations of LVEF measurement, including intra- and inter-operator variability [25] and hemodynamic load-dependence. When available, three-dimensional (3D) echocardiography should be preferred for LVEF assessment, as it provides greater accuracy and reproducibility [25]. However, its feasibility may be reduced in conditions that limit the acoustic window and the quality of ultrasound imaging [26]. It is the case for cancer patients who undergo concomitant radiotherapy (e.g., for breast cancer or lymphoma) and surgical interventions (e.g., mastectomy, breast implants/expanders). When image quality is suboptimal, the use of ultrasound contrast agents enhances the delineation of the endocardial border, improving the assessment of LVEF and LV volumes [27]. In contemporary cardiology, GLS has emerged as an earlier, more sensitive, and more reproducible marker of myocardial dysfunction than LVEF [4, 5], with strong evidence supporting its diagnostic and prognostic value also in cancer patients [28, 29]. A relative change in GLS has been proposed as an optimal parameter for detecting asymptomatic mild CTRCD [24, 30, 31]. Various threshold values have been explored over the last few years [32, 33]. Currently, a relative GLS reduction greater than 15% from baseline is the recommended threshold for the diagnosis of CTRCD, as this value seems to predict future significant decline in LVEF [29]. GLS measurements obtained by using 3D speckle-tracking echocardiography proved to have added value over two-dimensional (2D) speckle-tracking echocardiography in detecting cardiotoxicity [34]; however, its routine clinical use remains limited due to some critical constraints, including the need for optimal acoustic windows and high-quality images, specialized training, and the lack of standardization across vendors. LV myocardial work indices (MWIs), obtained from speckle-tracking derived strain parameters and non-invasive measured systolic blood pressure, have shown potential in improving the diagnosis and prognosis of CTRCD. A reduction in the global work index, particularly in patients with only a minor reduction in GLS but a significant decline in systolic blood pressure (~ 21 mmHg), may help identify patients at higher risk of CTRCD [35]. However, the optimal thresholds for the sequential use of GLS and MWIs require validation through large multicenter studies before clinical implementation.

The 2022 ESC guidelines on cardio-oncology [6] do not explicitly include LV diastolic dysfunction among the echocardiographic abnormalities for the diagnosis of CTRCD. Also, they do not provide specific recommendations on using diastolic dysfunction for initiating cardioprotective therapy or discontinuing cancer treatment. Nevertheless, evidence suggests that traditional indices of diastolic function [36] can also change during cancer treatment and could serve as early markers of subsequent systolic dysfunction, particularly in patients receiving anthracyclines and/or trastuzumab [31, 37, 38].

**Fig. 1:**
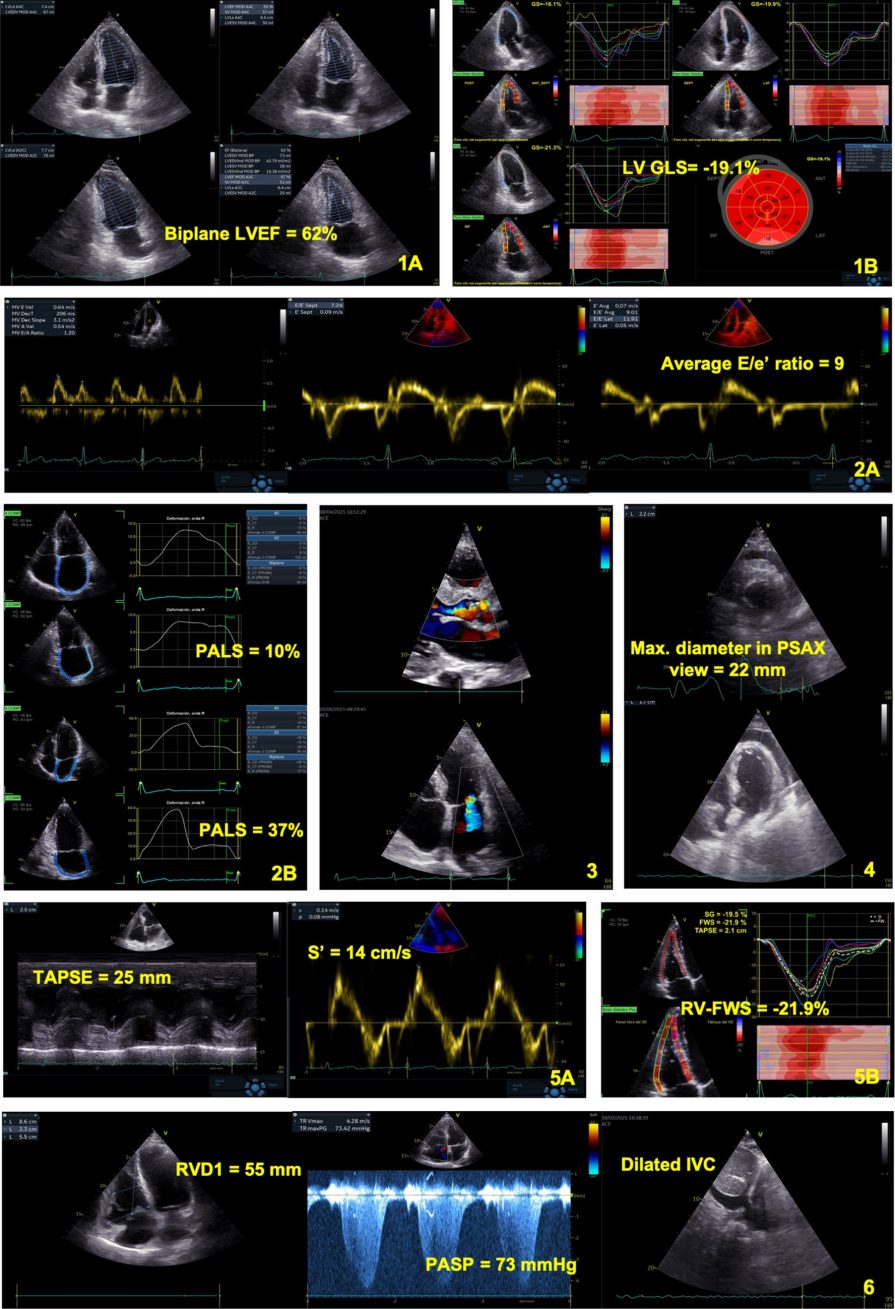
Key echocardiographic acquisitions during oncology patient’s evaluation. 1 Left ventricular systolic functionassessment by traditional Simpson biplane method (1A) and speckle tracking-derived global longitudinal strain (1B). 2Left ventricular diastolic function assessment by traditional methods (E/e′ ratio in 2 A) and left atrial strain (2B). 3 Aorticregurgitation (3A) and mitral regurgitation (3B) in patients undergone chest radiotherapy. 4 Right ventricular systolicfunction assessment by traditional methods (TAPSE and S′ wave velocity in 4 A) and speckle tracking echocardiography(4B). 5 Echocardiographic signs of pulmonary embolism in a cancer patient. 6 Pericardial effusion in a cancer patient

While the role of LV GLS in cardio-oncology is well characterized, little is known about the role of left atrial (LA) strain, which has been recently identified as a potential indicator of subclinical or early diastolic dysfunction [39, 40]. The STRANO study evaluated LA function using speckle-tracking echocardiography in women receiving chemotherapy for breast cancer. The results showed that LA strain impairment was significantly greater in patients who developed asymptomatic mild CTRCD [41]. Therefore, LA strain may serve as a promising parameter in this setting, warranting further validation. The prognostic significance and clinical utility of diastolic dysfunction remain poorly defined, highlighting the need for further research to clarify its relevance and potential implications in cardio-oncology. In Table 2. a checklist of the key echocardiographic parameters that should not be missing from an oncology patient’s report and their practical interpretation are reported. Complementary echocardiographic acquisitions are shown in Figure 1.

**Fig. 2:**
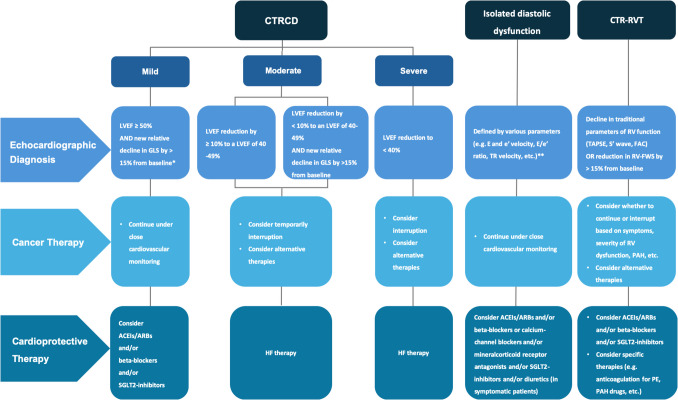
Diagnostic and therapeutic algorithm for the management of cardiac dysfunction induced by cancer therapy. ACEIs, angiotensin-converting enzyme inhibitors; ARBs, angiotensin II receptor blockers; CTRCD, cancer therapy-related cardiac dysfunction; CTR-RVT, cancer therapy-related right ventricular toxicity; FAC, fractional area change; GLS, global longitudinal strain; HF, heart failure; LA, left atrium; LAVi, left atrial volume index; LVEF, left ventricular ejection fraction; PAH, pulmonary arterial hypertension; PE, pulmonary embolism; RV, right ventricle; RV-FWS, right ventricular free wall strain; SGLT2, sodium–glucose cotransporter 2; TAPSE, tricuspid annular plane systolic excursion; TR, tricuspid regurgitation

The decision to initiate cardioprotective treatment or discontinue cancer therapy should be based on a comprehensive evaluation of multiple factors, including the severity of both cancer and CV symptoms, cancer prognosis, the availability of alternative cancer treatments, potential adverse drug reactions and interactions, and the patient’s preferences. A comprehensive and flexible diagnostic and therapeutic algorithm for the management of CTRCD, based on the integration of current guidelines and clinical experience, and potentially applicable across all classes of oncologic treatments, is proposed in Fig. 2. This flowchart integrates the different severity grades of CTRCD, including isolated diastolic dysfunction, and offers guidance on whether to continue or interrupt cancer treatment, as well as when to initiate cardioprotective therapy.

**Table 2:** Checklist of the key parameters to include in the echocardiographic report of oncology patients and their clinical implications

**Echocardiographic features**	**Clinical implications**
LV systolic function parameters • LVEF • LV GLS • LV MWIs (GMW and MWE)	LVEF and GLS reduction over time can indicate CTRCD MWIs may be measured for CTRCD risk stratification
LV diastolic function parameters • Traditional parameters (e.g., E/A ratio, septal and lateral e′ wave velocity, E/e′ ratio, TR velocity, etc.) • LA strain	Traditional parameters’ impairment over time can predict subsequent systolic dysfunction LA strain may be an early marker of diastolic dysfunction and an early predictor of CTRCD
RV systolic function parameters • Traditional parameters (TAPSE, S’ wave velocity, FAC) • 3D RVEF • RV-FWS and RV-GLS	Traditional parameters’ reduction over time can indicate CTR-RVT 3D RVEF may be measured for patient’s risk stratification Strain-derived parameters can be valuable markers of subclinical RV systolic dysfunction
Cardiac valves’ morphology and function	In patients undergoing RT, cardiac valves’ dysfunction my occur even years after the end of treatment
PASP	PASP increase during follow-up can be a sign of PE (TRPG usually ≤ 60 mmHg) or chronic PH
Arterial ventricular coupling (TAPSE/PASP ratio)	TAPSE/PASP ratio may be reported for risk stratification of patients with PE/PH
Pericardium	If present, pericardial effusion should be monitored over time, and possible signs of hemodynamic impairment should be looked for Pericardial thickening may be a sign of constrictive pericarditis
Pathology-specific ancillary findings • “McConnell sign,” right heart mobile thrombi, “60/60” sign • Septal bounce, “annulus reversus,” diastolic flow reversal in the hepatic veins • Intracardiac masses	PE Constrictive pericarditis Neoplastic lesions, thrombi or vegetations of infective endocarditis

CTRCD – Cancer Therapy Related Cardiac Dysfunction; CTR-RVT – Cancer Therapy Related – Right Ventricular Toxicity; FAC – Fractional Area Change; GLS – Global Longitudinal Strain; GMW – Global Myocardial Work; LAVi – Left Atrial Volume Index; LA – Left Atrium; LVEF – Left Ventricular Ejection Fraction; MWE – Myocardial Work Efficiency; MWIs – Myocardial Work Indices; PASP – Pulmonary Artery Systolic Pressure; PE – Pulmonary Embolism; PH – Pulmonary Hypertension; RT – Radiotherapy; RV – Right Ventricle; RVEF – Right Ventricular Ejection Fraction; RV-FWS – Right Ventricular Free Wall Strain; RV-GLS – Right Ventricular Global Longitudinal Strain; TAPSE – Tricuspid Annular Plane Systolic Excursion; TRPG – Tricuspid Regurgitation Pressure Gradient

### Right heart evaluation in patients undergoing cancer therapy: essential information for practice

Historically, studies on cancer-related toxicity have focused on the assessment of LV dysfunction. However, the impact of oncologic therapy on the right ventricle is increasingly recognized and has become the subject of growing clinical and scientific interest [42, 43]. A recent scientific statement by the HFA of the ESC and the ESC Council of Cardio-Oncology has proposed a new classification of cancer therapy-related right ventricular toxicity (CTR-RVT) based on two categories: symptomatic CTR-RVT, defined by right heart morpho-functional abnormalities with clinical signs and symptoms of right ventricular (RV) failure; asymptomatic CTR-RVT, defined as a > 15% reduction from baseline in RV free wall strain (RV-FWS) in the absence of significant impairment in other parameters, including 3D derived RV ejection fraction (Fig. 2) [44].

TTE represents the primary imaging modality for evaluating RV in cancer patients (Table 2, Fig. 1). Current cardio-oncology guidelines emphasize the importance of a baseline morphologic and functional evaluation of the RV in all oncologic patients referred for TTE. This recommendation is particularly relevant for patients with pre-existing conditions or at risk for RV dysfunction or scheduled to receive agents more frequently associated with RV and pulmonary circulation toxicity (e.g., anthracyclines, trastuzumab, cyclophosphamide, or dasatinib) [6, 45]. RV impairment typically develops concomitantly with LV dysfunction but may also occur in isolation [46]. Radiotherapy (RT) may contribute to RV dysfunction through direct cellular injury, amplified by the anatomical anterior position and thinner wall of the RV [42, 44].

The alteration of conventional parameters of RV systolic function during chemo-radiotherapy, including tricuspid annular plane systolic excursion (TAPSE), tricuspid annulus systolic velocity (S’ wave) obtained by tissue Doppler imaging, and fractional area change, is indicative of overt dysfunction, providing limited sensitivity for the early impairment [44]. Several studies showed a consistent decline in RV longitudinal strain parameters associated with cancer therapy, with more robust data about RV-FWS [47–49]. This impairment in the context of anthracycline-induced toxicity appears to exhibit a dose-dependent pattern [50]. Importantly, RV longitudinal strain values may decline during the early phase of CTR-RVT, when traditional echocardiographic parameters remain normal, being a valuable marker for subclinical dysfunction [42, 44]. In a cohort of 128 patients diagnosed with non-small cell lung cancer undergoing chemo-radiotherapy, significant alterations in both RV-GLS and RV-FWS were observed, despite no significant changes being observed in other conventional measures. Notably, RV-FWS demonstrated a correlation with 6-month all-cause mortality, underscoring its potential value as a prognostic marker [42]. Emerging evidence suggests that 3D strain may offer greater sensitivity for detecting RV subclinical dysfunction [46, 48]. Nonetheless, its routine application remains limited by the availability of specific software and technical challenges.

When feasible, 3D RV ejection fraction (RVEF) also provides incremental prognostic value over traditional 2D RV systolic function parameters [51] in patients undergoing cancer therapy. In a population treated with anthracyclines, Shen et al. [52] demonstrated that a 3D RVEF reduction was associated with worse outcomes, particularly when combined with an impaired LV GLS. However, similarly to conventional 2D parameters, a decrease in 3D RVEF may solely reflect a later stage of RV dysfunction.

As part of the right heart evaluation, it is important to routinely look for signs of RV acute and chronic overload. Pulmonary embolism (PE) is frequent in cancer patients due to several factors, such as the hypercoagulable state, surgical interventions, chemotherapy, and the use of central venous catheters [53]. TTE has limited negative predictive value in PE diagnosis, and the presence of specific echocardiographic signs, including the widely recognized “McConnell sign,” is relatively uncommon [54, 55]. Rarely, mobile thrombi “in transit” may be visualized in the right heart on TTE or transoesophageal echocardiography (TOE), a finding associated with a high mortality rate [56]. TTE remains a valuable tool for assessing hemodynamic instability secondary to PE and RV dysfunction, which, when present, is associated with worse prognosis [54, 57, 58]. In the context of acute PE, the non-adapted RV is unable to generate elevated pressures in response to an increased afterload; consequently, tricuspid regurgitation velocity (TRV) rarely exceeds 3.4 m/s, generating a tricuspid regurgitation pressure gradient (TRPG) ≤ 45 mmHg. Accordingly, the so-called 60/60 sign—defined as a pulmonary artery acceleration time ≤ 60 ms associated with a TRPG ≤ 60 mmHg—has been described [59].

Pulmonary hypertension (PH) is relatively common in cancer patients due to concomitant cardiopulmonary comorbidities, CTRCD, recurrent subclinical PE, or cancer-related pulmonary microangiopathy [60]. Furthermore, certain cancer treatments may contribute to PH development [44], such as dasatinib, which has been associated with pulmonary arterial hypertension (PAH) [55]. Current European guidelines on cardio-oncology recommend the discontinuation of dasatinib, even in asymptomatic patients, in cases of TRV exceeding 3.4 m/s or in the presence of confirmed PAH on right heart catheterization [6]. Radiation therapy and bleomycin may also lead to interstitial pulmonary fibrosis, potentially involving the pulmonary microvasculature and thereby increasing pulmonary vascular resistance [61]. Alkylating agents, interferon therapy, immune checkpoint inhibitors (ICI), and proteasome inhibitors have also been associated with the development of PH [62]. Moreover, surgical procedures such as lobectomy for lung cancer may result in an increased RV afterload attributable to a diminution of the pulmonary vascular bed [63]. In the case of chronic PH, signs of chronic RV pressure overload (e.g., subcostal RV free wall thickness > 5 mm and an enlarged right atrium in comparison to the left atrium) can be found [64], and TRV > 3.4 m/s (TRPG > 60 mmHg) is more frequently observed [65].

Recent studies have emphasized the prognostic value of arterial ventricular uncoupling, as assessed by the ratio between TAPSE and pulmonary artery systolic pressure (PASP) on echocardiography, in both PE and PH [66, 67]. A TAPSE/PASP ratio < 0.67 has been shown to be independently associated with increased all-cause mortality in patients with non-small cell lung cancer undergoing pulmonary resection [63]. Finally, echocardiography-derived pulmonary artery pulsatility index (PAPI) is emerging as a superior marker compared to TAPSE for predicting mortality in right heart failure conditions, including acute PE and PH [68, 69].

### Beyond function: echocardiographic assessment of the pericardium, valves, and cardiac masses

Pericardial involvement due to direct tumor infiltration, chemo-, or radiotherapy is a relatively frequent finding in cancer patients [70–72]. It is generally associated with advanced disease and is often indicative of a poor prognosis [71]. The treatment with ICI has been associated with a fourfold increased risk of pericardial involvement compared to patients not treated with ICI [73]. The most common manifestation is malignant pericardial effusion, which may evolve in some cases into cardiac tamponade [74]. TTE is the imaging modality of choice for the diagnosis and monitoring of pericardial effusion; it is critical for the detection of hemodynamic compromise in cardiac tamponade and is routinely employed to guide pericardiocentesis (Table 2, Fig. 1) [70, 75]. Constrictive pericarditis, characterized by pericardial thickening and impaired diastolic filling, also represents a possible, clinically significant manifestation of pericardial involvement in cancer patients [6, 71]. The most frequent etiologies include recurrent effusion and pericardial infiltration. Among patients previously exposed to thoracic radiotherapy, pericardial thickening has been reported in approximately 33% of cases at 20 years post-exposure [76]. Constrictive pericarditis may be suspected on TTE through certain hallmark features, including septal bounce, annulus reversus (lateral e′/medial e′ ratio < 1), diastolic flow reversal in the hepatic veins, reduced longitudinal strain in the ventricular free walls, and predominant impairment of radial strain [70, 71, 75, 77].

In cancer survivors, RT may lead to valvular heart disease (VHD), which can occur even decades after treatment (Fig. 1) [76, 78–80]. Valve injury is a dose-dependent process that starts with the thickening and fibrosis of the valve leaflets, and followed by subsequent calcific degeneration [80]. Left-sided valves are more commonly affected, likely due to their exposure to elevated pressure in comparison to the right-sided chambers. In populations assessed over more than 20 years following RT, the most prevalent VHD is aortic insufficiency, with a reported prevalence of 60%, followed by mitral insufficiency (52%), tricuspid insufficiency (26%), and aortic stenosis (16%) [76]. In contrast with the robust evidence supporting the association between RT and VHD, chemotherapy had long been considered solely a factor that could increase the risk of developing VHD [78]. However, certain studies showed an increased incidence of aortic and mitral degenerative VHD in patients who received anthracyclines, irrespective of RT exposure [81, 82]. In most cases, impairment of the aortic valve is parallel with ventricular dysfunction, suggesting that the two structures are concomitantly compromised [81]. Conversely, mitral insufficiency may also be a consequence of ventricular dysfunction induced by anthracyclines [81]. TTE serves as the primary diagnostic tool in the detection of cancer therapy-induced VHD. However, in case of uncertainty, TOE may prove useful to overcome issues related to suboptimal acoustic windows [83].

Finally, during routine echocardiographic surveillance in oncologic patients, incidental findings of intracardiac masses may occur, which encompass a wide differential diagnosis, including thrombi, vegetations, and neoplastic lesions (Table 2) [84]. Although TTE represents the first-line tool for detecting intracardiac masses, TOE is often needed for better resolution and anatomical detail, particularly in cases of suboptimal transthoracic acoustic windows. Cardiac metastases represent the most prevalent form of malignant cardiac involvement. Features suggestive of malignancy include localization in the right heart chambers, irregular or lobulated borders, heterogeneous echogenicity, sessile attachment, progressive growth over time, coexistence of pericardial effusion, and evidence of infiltration of surrounding structures [85]. Myocardial infiltration may also manifest as regional impairment of myocardial strain or focal hypo-akinesia [86]. Of note, diffuse myocardial infiltration due to metastases is more commonly observed in lymphomas, sarcomas, and melanomas [87]. Cancer-related hypercoagulability predisposes patients to intracardiac thrombus formation [47]. LV thrombi are frequently associated with CTRCD with reduced LVEF [47], whereas right-sided thrombi are commonly linked to the presence of central venous catheters or device leads [47]. Contrast-enhanced echocardiography can improve visualization in suboptimal acoustic windows and assist in differentiating thrombi from malignant masses, as the latter typically exhibit contrast enhancement due to vascularization [88]. Infective endocarditis should be considered in the differential diagnosis of mobile, irregular intracardiac masses [84]. Cancer patients are at heightened risk due to both immunosuppression, either related to disease or treatment, and the frequent use of central venous catheters [89].

### Advanced imaging modalities in cardio-oncology: beyond echocardiography

Although echocardiography remains the primary imaging modality owing to its wide availability, cost-effectiveness, and real-time functional assessment capabilities, it has certain limitations. Cardiac magnetic resonance (CMR) is considered the gold standard for the assessment of cardiac chamber volumes, myocardial mass, and contractile function [90] and, beyond functional evaluation, offers advanced tissue characterization, strain imaging, and assessment of myocardial perfusion. In cardio-oncology, CMR is mainly used as a second-line diagnostic modality after echocardiography, particularly in patients with suboptimal acoustic windows due to obesity, pulmonary disease, chest wall abnormalities, or breast implants [6]. Moreover, current guidelines on cardio-oncology recommend CMR when ICI myocarditis is suspected both for diagnosis and subsequent monitoring during therapy [6, 91]. CMR is also the reference standard for detecting pericardial involvement, due to its superior characterization of pericardial thickness, composition, and restrictive physiology, and is valuable in differentiating hemorrhagic from serous effusions [70, 92]. Finally, CMR remains the gold standard for the evaluation of intracardiac masses, owing to its superior capacity for tissue characterization, assessment of myocardial infiltration, and delineation of anatomical relationships [6]. In the context of pericardial involvement, computed tomography is the reference imaging modality for the identification of pericardial calcifications [70, 92]. Finally, cardiac computed tomography angiography (CCTA) represents a useful tool for the identification of atherosclerotic CV disease. Oncological patients face an increased bleeding risk, making the utilization of a non-invasive tool as CCTA a particularly beneficial approach in this population [93]. CMR and computed tomography findings and indications in cancer patients can be found in Table S2, Supplementary Material.

### Cardiac imaging surveillance and follow-up during cancer therapy

Structured surveillance protocols are required to ensure the timely detection of asymptomatic cancer therapy-induced cardiotoxicity and to guide the initiation of appropriate cardioprotective strategies before there’s a significant decline in cardiac function, thus avoiding oncology treatment’s interruption. Longitudinal cardiac imaging monitoring should be tailored to the patient’s baseline CV risk profile, the oncologic regimen, and the presence of comorbidities. The frequency of follow-up is therefore directly related to the estimated risk of cardiotoxicity. TTE, including advanced techniques (e.g., 3D and speckle-tracking echocardiography), is the preferred imaging modality to monitor cancer patients during therapy. The 2022 European Society of Cardiology (ESC) guidelines on cardio-oncology [6] recommend surveillance protocols that detail the timing of echocardiographic examinations based on the patient’s cardiovascular risk and oncology treatment regimen. An in-depth analysis of these protocols is beyond the scope of this review.

## Conclusions

In the evolving field of cardio-oncology, TTE remains a cornerstone for CTRCD diagnosis and monitoring. The integration of traditional echocardiographic parameters and advanced imaging techniques allowed early detection of subclinical myocardial damage and improved risk stratification of cancer patients. Further validation in larger prospective studies is needed to define the prognostic significance of novel ultrasound tools, including speckle-tracking and 3D-echocardiography, and their therapeutic implications in cardio-oncology. Close collaboration between cardiologists and oncologists is essential to ensure that all cancer patients, including those at high and very high CV risk, have access to the best possible treatment, minimizing the risk of cardiotoxicity and initiating cardioprotective therapy in a timely manner where necessary.

## Supplementary Information

Below is the link to the electronic supplementary material.

ESM1 (DOCX.88.5 KB)

## Data Availability

No datasets were generated or analysed during the current study.

## Abbreviations

2D: Two-dimensional
3D: Three-dimensional
CCTA: Cardiac computed tomography angiography
CMR: Cardiac magnetic resonance
CTRCD: Cancer therapy-related cardiac dysfunction
CTR-RVT: Cancer therapy-related right ventricular toxicity
CV: Cardio-vascular
ESC: European Society of Cardiology
GLS: Global longitudinal strain
HER2: Human epidermal growth factor receptor 2
HF: Heart failure
HFA: Heart Failure Association
ICI: Immune checkpoint inhibitors
IC-OS: International Cardio-Oncology Society
LA: Left atrial
LV: Left ventricular
LVEF: Left ventricular ejection fraction
MWI: Myocardial work index
PAH: Pulmonary arterial hypertension
PAPI: Pulmonary artery pulsatility index
PASP: Pulmonary artery systolic pressure
PE: Pulmonary embolism
PH: Pulmonary hypertension
RT: Radiotherapy
RV: Right ventricular
RVEF: Right ventricular ejection fraction
RV-FWS: Right ventricular free wall strain
TAPSE: Tricuspid annular plane systolic excursion
TOE: Trans-oesophageal echocardiography
TRPG: Tricuspid regurgitation pressure gradient
TRV: Tricuspid regurgitation velocity
TTE: Trans-thoracic echocardiography
VHD: Valvular heart disease
