# Supplementary material for: Echocardiographic monitoring in cancer therapy: clinical guidance for cardiologists and oncologists
Source: Heart Fail Rev. 2025 Oct 24;30(6):1591–602. doi: 10.1007/s10741-025-10569-0 (PMC12618327; doi:10.1007/s10741-025-10569-0)
Supplement: Supplementary file 1 — (DOCX.88.5 KB) [file 10741_2025_10569_MOESM1_ESM.docx]

**Table S1** Baseline cardiotoxicity risk stratification*

| **Baseline CV disease/risk factors** | **Anthracycline chemotherapy** | **HER2-targeted therapies** | **VEGF inhibitors** | **BCR-ABL**  **inhibitors** | **Multiple myeloma**  **therapies** | **RAF and MEK**  **inhibitors** |
| --- | --- | --- | --- | --- | --- | --- |
| HF/cardiomyopathy/CTRCD | Very high | Very high | Very high | High | Very high | Very high |
| Severe VHD | High | High | - | - | - | High |
| Ischemic disease (stable angina, MI, previous PCI or CABG) | High | High | Very high | - | - | High |
| Arterial vascular disease | - | - | Very high | Very high | Very high | - |
| PH | - | - | - | High | - | - |
| DVT/PE | - | - | High | Medium2 | Very high | - |
| Atrial fibrillation/flutter, ventricular tachycardia/fibrillation | - | Medium2 | Medium2 | Medium2 | Medium2 | Medium1 |
| Arterial hypertension | Medium1 | Medium1 | High | Medium2 | Medium1 | Medium2 |
| CKD (eGFR < 60 ml/min/1.73m^2^) | Medium1 | Medium1 | Medium1 | Medium1 | Medium1 | Medium1 |
| Diabetes mellitus | Medium1 | Medium1 | Medium1 | Medium1 | Medium1 | Medium1 |
| Hyperlipidaemia | - | - | Medium1 | Medium1 | Medium1 | - |
| Current smoker | Medium1 | Medium1 | High | Medium1 | Medium1 | Medium1 |
| Obesity (BMI > 30 Kg/m^2^) | Medium1 | Medium1 | Medium1 | Medium1 | Medium1 | Medium1 |

*The table was adapted from 2022 ESC Guidelines on cardio-oncology developed in collaboration with the European Hematology Association (EHA), the European Society for Therapeutic Radiology and Oncology (ESTRO) and the International Cardio-Oncology Society (IC-OS).

BCR-ABL – breakpoint cluster region-Abelson oncogene locus; CABG – coronary artery bypass grafting; CKD – chronic kidney disease; CTRCD – cancer therapy related cardiac dysfunction; CV – cardiovascular; DVT – deep vein thrombosis; HER2 – human epidermal receptor 2; HF – heart failure; MEK – mitogen-activated extracellular signal-regulated kinase; MI – myocardial infarction); PCI – percutaneous coronary intervention; PE – pulmonary embolism; PH – pulmonary hypertension; RAF – rapidly accelerated fibrosarcoma; VEGF – vascular endothelial growth factor; VHD – valvular heart disease.

Risk level:

- Low risk is defined by no risk factors OR one Medium1 risk factor;

- Medium risk is defined by medium risk factors with a total of 2-4 points

- High risk is defined by any high-risk factor OR medium risk factors with a total ≥ 5 points;

- Very high risk is defined by any very high-risk factor.

Medium1 = 1 point.

Medium2 = 2 points.

**Table S2** Cardiac magnetic resonance and computed tomography use in cancer patients

| **Imaging modality** | **Parameters** | **Indications** |
| --- | --- | --- |
| CMR | - LV mass, volumes and ejection fraction - RV volumes and ejection fraction - Longitudinal and circumferential strain (when available) - Myocardial characterization by T2w (STIR), T1, T2 maps, LGE - Myocardial perfusion - Intracardiac masses - Pericardium | - Assessment of cardiac function in the presence of poor-quality echocardiography windows or non-diagnostic echocardiography - Diagnosis and monitoring of myocarditis (e.g. suspected ICI-associated myocarditis)/ cardiomyopathy (e.g. AL-CA)/Takotsubo syndrome - Clinical suspicion of coronary artery disease (perfusion CMR) - Tissue characterization of intracardiac masses and delineation of their anatomical relationships/detection of intracardiac thrombi - Detection of pericardial inflammation and constrictive physiology/characterization of pericardial effusion (hemorrhagic vs serous) |
| CT | - Coronary arteries (CCTA) - Pericardium - Intracardiac masses | - Rule out of obstructive coronary artery disease/ Takotsubo syndrome - Identification of pericardial calcifications - Differential diagnosis between malignant and benign lesions (together with PET) |

AL-CA – amyloid light-chain cardiac amyloidosis; CCTA – coronary computed tomography angiography; CMR – cardiac magnetic resonance; CT – computed tomography; ICI – immune checkpoint inhibitors; LGE – late gadolinium enhancement; LV – left ventricular; PET – positron emission tomography; RV – right ventricular; STIR – short tau inversion recovery.
